# Genome Sequence of *Enterobacter turicensis* Strain 610/05 (LMG 23731), Isolated from Fruit Powder

**DOI:** 10.1128/genomeA.00996-13

**Published:** 2013-12-05

**Authors:** Roger Stephan, Christopher J. Grim, Gopal R. Gopinath, Mark K. Mammel, Venugopal Sathyamoorthy, Larisa H. Trach, Hannah R. Chase, Séamus Fanning, Ben D. Tall

**Affiliations:** Institute for Food Safety and Hygiene, University of Zurich, Zürich, Switzerlanda; CFSAN, FDA, Laurel, Maryland, USAb; UCD Centre for Food Safety, School of Public Health, Physiotherapy & Population Science, University College, Dublin, & WHO Collaborating Centre for *Cronobacter*, Belfield, Dublin, Irelandc

## Abstract

We report the draft genome sequence of *Enterobacter turicensis* strain 610/05 (LMG 23731), isolated from fruit powder. The draft genome has a size of 4,182,790 bp and a G+C% content of 58.0.

## GENOME ANNOUNCEMENT

Stephan et al. (1) reported the isolation from fruit powders of 12 bacterial strains, which were presumptively identified as *Enterobacter sakazakii* (now genus *Cronobacter*) based on colony appearances on *Cronobacter* chromogenic agars. API32E analysis revealed that these isolates were not *Cronobacter* and also that they did not match any existing identification profiles with high confidence. Using a polyphasic analysis scheme, Stephan et al. (1) proposed to classify two of these strains as the novel species *Enterobacter turicensis*. Interestingly, *Cronobacter* comprises a diverse genus of pathogens that cause life-threatening infantile infections, such as brain abscesses, meningitis, necrotizing enterocolitis, and septicemia (2, 3). There is, however, no indication that *E. turicensis* is pathogenic to humans. Not surprisingly, *E. turicensis* was found to share several physiological traits with the genus *Cronobacter*, such as production of a yellow, *Pantoea*-like carotenoid pigment (4) and constitutive metabolism of 5-bromo-4-chloro-3-indolyl-a-d-glucopyranoside, two traits used in the differentiation of presumptive *Cronobacter* colonies.

Recently, Brady et al. (5) proposed that *E. turicensis* be recognized as a new *Cronobacter* species and subsequently, Masood et al. published the draft genome sequence for the type strain of *E. turicensis*, 508/05 (LMG 23730; DSM 18397) (6). Because the taxonomic position of these strains remains unclear, we sequenced *E. turicensis* strain 610/05 (LMG 23731). A library was constructed using a Nextera XT DNA sample preparation kit (Illumina, San Diego, CA), and whole-genome sequencing was performed on a MiSeq benchtop sequencer (Illumina, San Diego, CA), utilizing 500 cycles paired-end version 2 chemistry. FASTQ datasets were trimmed and assembled using default parameters in CLC Genomics Workbench, version 6.0.5 (CLC bio, Aarhus, Denmark). The draft genome of strain 610/05 is 4,182,790 bp, on 262 contigs (>500 bp in size), and has a G+C% content of 58.0. Genomic contigs were annotated using the RAST annotation server (7) to identify RNAs and protein-coding genes. The draft genome of strain 610/05 is predicted to contain 3,857 coding sequences (CDS).

The *E. turicensis* strain 610/05 draft genome sequence was highly clonal with that of strain LMG 23730^T^ (average nucleotide identity of 99.98), as reported by Masood et al. (6). The genome possessed a number of noteworthy features, including six chaperone-usher fimbriae; curli, dulcitol, and malonate utilization; and a type III secretion system gene cluster similar in gene content to those of *Pectobacterium carotovorum* and *Pseudomonas syringae*. The genome contained two conjugative plasmids, an IncF (*tra*) plasmid that contains a copper homeostasis operon and an IncN (*virB*) plasmid similar to that found in *Salmonella enteritidis* serovar Agona strain SL483. It should be noted that the IncF plasmid was not homologous to the common virulence plasmid reported among *Cronobacter* spp., which contains a number of genus- and species-specific virulence factors (8).

### Nucleotide sequence accession numbers.

The whole-genome shotgun project for *E. turicensis* strain 610/05 is available in GenBank under accession number AXDM00000000. The corresponding NCBI Biosample record SAM02319257 is subject to taxonomic revision.
